# Role of higher education system in promoting law abiding behavior among students

**DOI:** 10.3389/fpsyg.2022.1036991

**Published:** 2022-11-17

**Authors:** Yan Dong, Sadaf Zeb

**Affiliations:** ^1^Law School, Tianjin University, Tianjin, China; ^2^Psychology Department, Capital University of Science and Technology, Islamabad, Pakistan

**Keywords:** legal socialization, legal education, moral education, law abiding behavior, psychological factors, higher education

## Abstract

University phase is a major turning point in youth's life and this is a time of profound mental and cognitive development of students. Without proper direction and guidance, it is common for students to develop deviant behaviors, non-law abidance and unhealthy beliefs. In this regard, an integral part of the educational process is the imparting of moral values and law-abiding behaviors in students. The objective of this study was to explore the role of higher education system in fostering law-abiding behavior among Chinese students, as well as the issues it causes for society. For this purpose, the principles of law-abiding behavior were studied and key psychological factors used in the system were identified. The suggestions of the bibliometric research are designed to improve and expand the method for preventing student misbehavior in educational institutions, hence enhancing the efficacy of preventative work with students. They may serve as the basis for the creation and enhancement of programs and strategies aimed at teaching lawful behavior among students. More than 3,785 articles were published related law-abiding behavior from 2000 to the end of July 2022 years were examined in this research using the Scopus database and the original sample was narrowed down to include only articles, book chapter and conference papers that contributed to law-abiding behavior and higher education literature. The VOS viewer software was used to execute the descriptive statistics and scientific mapping approaches using co-citation analysis. In the descriptive analysis, we analyzed publishing patterns over time, the geographical localization of the contributing institutions, journals, the most prolific authors. The findings of the present study may also provide the foundation for a planned educational initiative whose ultimate aim is to produce a fully realized, harmonious, self-reliant, mature, and law-abiding person. The study has provided supporting evidence for how youngsters legal sensibilities are shaped in universities have been implemented. Two distinct but interdependent educational spheres, the normative legal sphere and the space for the creation and development of students' personalities must work together to raise and educate youngsters.

## Introduction

Law-abiding behavior is stated as a person's consistent adherence to the most important societal rules, his efforts to maintain public order and equilibrium, and his preservation of individuality. Higher education institutes playa a vital role in promoting law abiding behavior in students and refers to level of education that is offered by high schools and other educational institutions (Niu, 2022). It also plays a significant part in the economic and social advancement of individuals (Popova and Popovs, 2022). The primary purpose of higher education is to generate and disseminate knowledge for the benefit of individuals and communities (Ramaswamy et al., 2021; Haniya and Said, 2022). Besides that, education serves to elevate individuals from ignorant conditions. Through education, everyone should be made aware of their rights and responsibilities in order to enlighten the populace and improve the insufficient social conditions. Because of the growing number of young criminals in today's society, it's important that legal literacy permeate all levels of society. Therefore, law-related common knowledge and responsibility should be communicated to students as early as feasible, especially those at the grassroots level, to reduce the likelihood of students making irrational decisions (Jiyan, 2020).

Since universities play a pivotal role in the development of future leaders, teaching students about the rule of law is a crucial component of rule of law education at the national level. The rule of law is a social institution that establishes norms for the behavior of all members of society within a given state. Despite the importance of laws in maintaining peace and order in society, some people, especially today's youth, choose to ignore them (Ulfah et al., 2021). The formation of a rule-of-law society, the advancement of modernization, and the growth of rule of law education for university students are all dependent on the growth of rule of law education for university students (Li et al., 2022). For instance, previous research has shown that increasing one's level of education has a constructive effect on the maturation of one's moral reasoning. In addition, the progress in education especially higher education, is a shared responsibility among all components, including students. Because students' time at university marks a significant turning point in their lives (Ran et al., 2022) and are not just expected to be academically successful while also displaying the kind of character qualities that can steer the country in a more positive path. Likewise, character education is the best course of action for the next generation to find their way and avoid destructive habits (Dewantara et al., 2021).

The significance of education, morality, human responsibility, the capacity to collaborate and compete, and the capacity to make independent decisions regarding the selection of diverse behavioral tactics are all highly valued by society. A person's social and legal activities, as well as his willingness to contribute to establishing the rule of law and order, depend on his legal and civil competence, which is an essential component of his general culture (Maltseva et al., 2020). The obedience to the law is obedience that originates with public knowledge of an existing law. Besides that, legal consciousness is a belief in the legal values inherent in humans. Moreover, institutional law such as that found in university, is just as important as the laws enacted by society and the government (Haitao, 2022). Due to the lack of effective rules, new types of rights violations always appear as technology advances. The purpose of legal education is to instil in individuals an appreciation for the value and significance of the law, as well as an adherence to it. However, some people might intentionally break the law due to ignorance of legal norms, while others could violate the law despite being aware of it, leading to major disruption in society (Jiyan, 2020). The lack of legal literacy and the low legal standard among Chinese university students make it impossible for the country to build a rule-of-law society or implement the market-based economic reforms that are necessary for sustained economic growth. Students are influenced by the hostile social environment and as a result (Han et al., 2022), they do not follow the rules and do not regret their illegal behavior. They are willing to take chances and try the law on their own to settle disputes and conflicts (Li et al., 2022). Institutions of higher education play an important role in the process of legal socialization, which is how young people “grow their relationship with the law by learning law-related values, attitudes, and reasoning skills” (Naftali, 2022).

Law-abiding behavior “works” because people have control over themselves. The “law-abiding behavior” encompasses a harmony between adopting and adhering to social norms and between one's own sense of duty and responsibility and one's actual action (Maltseva et al., 2020). Since the 18th Communist Party of China (CPC), the General Secretary of CPC has put a lot of effort into promoting and educating people about the rule of law (Liu, 2020), making people more aware of the need to follow the law, and boosting the cultural development of socialist rule of law. He has made a number of remarks, including “insisting on the popularization of the law and compliance with the law for all people as a long-term core job of the rule of law” and “keeping up with the times in the popularization of the law and making intensified efforts on the relevance and efficacy,” which have clarified the fundamental stance, key tasks, and essential practices in the publicity and education of law (Guan et al., 2022). As an example, China's government adopted market reforms and an Open-Door policy in 1978, following nearly 30 years of a command economy and revolutionary struggle. Over the past 40 years or more, the government of the People's Republic of China has worked to reconstruct the country's legal system. Along with changes to the legal system, the government started large-scale information and education campaigns to teach officials and citizens about the new laws and the concept of fazhi, which can be translated as “legal rule” or, more controversially, “rule of law” (Naftali, 2022). Additionally, there are two components of legal consciousness: legal ideology and legal psychology. In this context, “legal ideology” refers to a body of thought that offers a unifying theoretical framework for analyzing a wide variety of legal phenomena. While emotional comprehension of legal issues is what legal psychology is all about. For instance, if young individuals are aware of the law and can envision it, but are unresponsive to legal directives, it is evident that their legal consciousness is not completely developed (Yakubov, 2022).

The significance of current study is to explore the role of higher education system in promoting law abiding behavior in Chinese students. Due to rapid development of reform in our nation, the market economy has grown increasingly active, and the application of norms in social life has become increasingly prevalent. The rule of law is crucial because students are the future of the country and a key to its revival (Xu, 2022). In this regard, the formation of law-abiding behavior, the identification and eradication of causes and situations that lead to the development of deviant behavior is one of the main areas of action for all subjects of the preventative system, including bodies exercising control in the field of education and institutions engaged in educational activities (Salakhova et al., 2020). Therefore, the purpose of this study was to investigate the influence of higher education institutions in fostering law-abiding behavior among Chinese students. The study also focused on how different psychological factors, such as age, gender, education, etc., impact Chinese students' lawfulness. This study contributes to the significance of law-abiding behavior of students in higher education institution and also encompasses the cultivation of students' ethical, moral, legal, and psychological wellbeing. Moreover, in order for students to develop a healthy sense of self, it is their responsibility to learn ethical management skills and to be taught the right values and worldview. Thus, students need to have national self-esteem, self-confidence, and pride; establish an accurate viewpoint on life, values, and the world; have a sound personality in the long term for success. In the past, few studies have been conducted in this field.

### Research questions

1. What is law-abiding behavior?
2. What factors affect the higher education student's awareness of law-abiding in China?
3. Which factors have more significant impact on law abiding of higher education students?
4. Is there any relationship between law abiding behavior and psychological factors in higher education students?

### Research objectives

1. To define the law-abiding behavior.
2. To examine the factors that impact law abiding behavior.
3. To find out the significant differences on gender, age education, occupation and location with law abiding among higher education students.
4. To examine the relationship between law abiding behavior and psychological factors in higher education students.

## Literature review

### Psychological factors and law-abiding behavior

Young people's legal education is the organization of educational activities by higher education institutions to assess the knowledge of aspiring legal professionals (Ramaswamy et al., 2021), awareness, and culture of the law in order to better inform them of their own rights and obligations as individuals, the nature of law as it pertains to society and humanity, and the importance of entrepreneurship in the legal field. Aside from that, the educational part is the basis for how a young person's personality develops and changes, as well as how standards and guidelines for behavior are set (Haider et al., 2022). The rule of law depends on people obeying the rules set forth and enforced by the government. Contemporary debates about lawfulness revolve around the notion that people will be less likely to disobey the law if they are aware they will be caught and punished. As people in a law-abiding society don not act out of fear, but because they want to act in a socially acceptable and moral way. A society like this is self-regulatory because its people take it upon themselves to follow the law. So, people in a morally driven society voluntarily obey the law and the people in charge of it because they believe: that the things that are against the law are also wrong and that the people in charge of the law have the right to be obeyed (Tyler and Darley, 1999). However, there is a dearth of research that investigates the role of higher education in shaping students' moral identities (Kozorez et al., 2022).

In China, the government doesnot try to hide the fact that they use surveillance tools, face recognition systems, biological identifiers, and social networks to find out accurate information about each citizen. They call this an important step toward e-government because it makes more people obey the law (Alguliyev and Alakbarova, 2021). According to the prior literature, over 30% of cyclists do not use helmet because it's too hot, and nearly 20% don't wear helmet because they are a hard to store. In spite of the importance of e-bike helmet rules and regulations, there has been a dearth of research on the topic, especially in China (Zhou et al., 2022).

According to the Law “On Education in the Chinese Federation”, students should engage in character-building activities that promote their own growth and the formation of an environment where they can exercise autonomy and socialize with others. Education is a way for people to build their character and create their own culture. During the learning process, students develop perspectives, a scientific worldview, an understanding of the laws of nature, society, and thinking, moral and aesthetic ideas, as well as the ability to conform to social norms and obey its laws (Kozorez et al., 2022). In the same way, it would be important to look at how law-abiding helps the students' wellbeing, since non-normative political involvement, like not obeying the law, is bad for one's wellbeing. Since academic dishonesty like plagiarism is on the rise in the university sector, students' compliance with the law is especially important (Shek et al., 2022). In addition, institutions of higher education play a crucial role in establishing the rule of law. In this way, education systems that support and teach respect for the rule of law in line with international human rights and basic freedoms build trust between students and public institutions. Teaching based on the rule of law can help students become more independent thinkers who have a firm grasp on the concepts of responsibility, fairness, and equality (Li and Sun, 2022). According to earlier research, teaching anti-corruption to students is crucial to the success of character education, as it helps them recognize the ways in which corrupt behavior can have negative consequences for the individual, the state, and the nation (Dewantara et al., 2021). Similarly, prior research has discovered a negative correlation between academic dishonesty and factors including intrinsic motivation, self-efficacy, utilitarian value, and internal locus of control in both the classroom and the wider world (Malesky et al., 2022). There is a growing body of research linking certain student attitudes, behaviors, or character attributes to dishonesty in the classroom. Researchers, for instance, have discovered that a student's mindset is a major contributor to academic dishonesty (Yu et al., 2021). Moreover, previous research has also shown that teaching law helps individuals develop a respect for the rule of law and an appreciation for personal accountability (Jiyan, 2020). The aim of the present study was to explore the influence of various psychological factors on law abiding behavior.

#### Law abiding behavior and gender

A person's law-abiding conduct is consistent when he or she abides by the most important social rules, works to keep the peace and harmony in society, and yet maintains their own unique identity. People's ability to establish self-control is what makes lawful action successful. Thus, “law-abiding behavior” encompasses not only the acceptance and observance of societal standards, but also the preservation of an internal harmony between a sense of duty and responsibility and one's actual behavior. It is important to remember that all actions are based on patterns from the past. The process of establishing a learned behavior typically involves imitation. The educational system is the primary institution for the socialization of young people, where one's personality, values, attitudes, rules of conduct, and legal competence are formed in accordance with generally accepted moral standards and the current conditions of social development. Consequently, educational institutions support the transition of youngsters from the period of imitating acceptable conduct to the stage of becoming a law-abiding person through the provision of a range of curricular activities. Education, morality, personal responsibility, cooperation, competitiveness, and the capacity to think critically and make autonomous judgments about one's actions are all attributes that are held to very high standards in today's society. A person's social and legal activity, as well as his or her desire to contribute to establishing the rule of law and order, rely on his or her legal and civil competence, which is an important component of his or her general culture. For instance, a number of studies have found that women tend to be more ethically sensitive than men (Dkadek, 2022). This shows that women were more sensitive to law abiding behavior as compared to men. A meta-analysis of research into gender differences in cheating found that women were more likely to view cheating negatively than men. In particular, women are viewed as more ethically sensitive, rule-abiding, and concerned about the consequences of their actions because of the emphasis they place on relationships and care for others in their moral reasoning. Men are more likely to be individualistic, competitive, and risk-taking, and their moral reasoning is based on a sense of justice and a desire for personal success (Zhang et al., 2018).

#### Law abiding behavior and age

University students' propensity for lawful activity may be influenced by a number of psychological factors, including their age. One of the strongest correlates of criminal activity is age. The total number of offences rises until late adolescence, then drops dramatically thereafter. This pattern is very consistent across different time periods, cultures, groups at risk, and types of crimes (Tomczyk et al., 2020). According to previous literature, it was found that as with age and when one get more professional experience, they become more sensitive to ethical issues. Because of this, today's youth often lack the moral convictions of their elders. Along these lines, numerous studies have found that elderly persons generally have more developed moral principles than their younger counterparts (Dkadek, 2022). Similarly, in a prior study, for instance, the helmet-wearing rate was considerably higher among those aged 40 and older compared to those aged 25–40 and 16–24 (Sharif et al., 2022).

#### Law abiding behavior and educational background

Education has also played an important role in molding the attitudes, values, and beliefs of individuals. In particular, education makes people see the world more clearly. Besides that, the significance of legal education, which is the process of influencing an individual with an organized, methodical, clear objective, which produces legal consciousness, legal instructions, law-abiding behavioral abilities and habits. For instance, previous research has indicated that those with more education tend to have more egalitarian attitudes on gender roles than people with less education, showing that education may alter people's perspectives (Du et al., 2021). Likewise, studies have shown that people with a college degree or higher have a much deeper understanding of the rules regarding helmet use. Moreover, previous research has shown that people with higher levels of education are more likely to wear helmets while cycling (Sharif et al., 2022). People with higher levels of education are more likely to respect authority figures and take accountability for their own actions (Wu et al., 2022). The rates of youth violence, criminality, child abuse, domestic violence, and absentee parenting have all been steadily declining over the last several years. However, the relevance of the operations of social institutions to avoid negative social phenomena is not diminished by the specifics of the offences themselves, the lowering of age limitations of offenders, or the rising share of youth violence. Because universities have such a significant impact on shaping the character of children and young adults, they are a good place to start when designing a strategy to reduce youth criminality. To address this issue, we need to design and execute strategies for universities to use with youngsters to help them grow into law-abiding citizens. Programs and approaches of this kind should be all-encompassing and systemic, guaranteeing the use of psychological and classroom practices techniques aimed at helping youngsters grow into healthy adults with strong character traits like the ability to reflect on their own actions and learn from their mistakes, an understanding of the law, and the foundational skills necessary for achieving personal fulfilment. Furthermore, the substance of the processes of the normal mental development at each age stage must be considered in the system of shaping law-abiding conduct in educational institutions for youngsters. Psychological and classroom practices work with youngsters in groups that provide educational activities might benefit from including the age-psychological approach with the system-activity approach as its methodological foundation.

#### Law abiding behavior and occupation

An individual's behavior consists of the thoughts, feelings, and deeds that are intrinsic to that individual. If a person is taught right from wrong at a young age, they will be much more likely to continue to exhibit those values as adults. Especially when it comes to excellent conduct in business or decisions that affect one's professional ethics. Where in business is very important (good) behavior for the long-term success of a business and for relationships between people? Behaviors need to be taught. People who are used to acting badly in business will always cheat in business. Honesty and responsibility are two virtues that should be inculcated. Where being honest is one of the most important business skills. Effective working relationships between employees, colleagues, and customers. Business activities are affected by responsible conduct, such as preventing plagiarism (Haitao, 2022). Prior research revealed that self-employed cyclists had the highest percentage of helmet use, followed by students and the unemployed (Sharif et al., 2022). According to prior research, legal professionals are more inclined than others to obey the law.

#### Law abiding behavior and location

There is a significant association between law abiding behavior and location. Previous research has shown that city centers had a higher helmet use rate than macro-centers and peripheral places. A similar finding was made in another study conducted in China, which found that helmet use was significantly higher on city roads than on provincial roads, country roads, or national highways. Further, in a cross-sectional study, the number of drivers who did not wear a helmet was higher on highways and roads outside of town than on the main roads (Sharif et al., 2022).

## Research methodology

**Figure 1:**
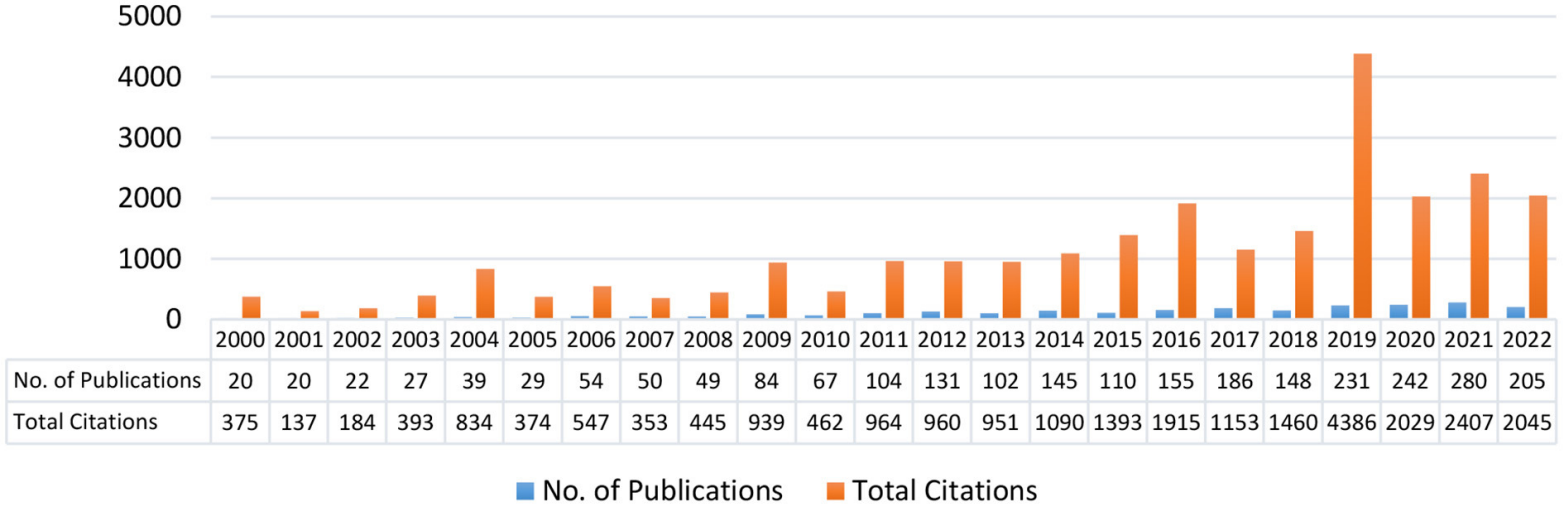
Number of articles published in the period 2000 till end of July 2022.

Bibliometrics is a research strategy that use statistical reviews of previously published scientific articles, books, conference articles in order to evaluate the significance of publications and offer an overview of the existing body of knowledge in a certain area of study (Grosseck et al., 2019). Bibliometric research is useful because its micro-level analysis may be used to identify patterns and trends within a limited subject area (Mani et al., 2022). However, it is important to remember that bibliometric analysis is not appropriate for comparing individual researchers or research teams. In addition, bibliometric research may take many forms, such as journal rankings, evaluations of research quality, surveys of published works, and analyses of patterns and trends (Hammarfelt and De Rijcke, 2015). This study follows the framework of a previous article that examines trends and patterns in the higher education sector in an effort to map out a roadmap for future orientations within the context of Law-abiding behavior. In this work, the methodology incorporates three major steps: (1) data gathering, (2) data cleaning, and (3) bibliometric analysis. Figure 1 illustrates an overview of the procedure.

Using the Scopus database, this research compiled a list of articles on the use of Law-abiding behavior in the higher education sector (Ghani et al., 2022). Scopus database is one of the most widely used citation databases, making it a great resource for researchers interested in bibliometric study of higher education sector. Due to the lack of literature on the topic of Law-abiding behavior in the higher education sector, all works published in English were included in this investigation. This included peer-reviewed academic journal articles, book chapters, and conference proceedings. The data was gathered in July 2022. In order to find relevant results, we mixed Boolean operators with specified phrases (i.e. AND, OR). Starting with broad keywords (Law abiding behavior AND Higher Education OR China OR Gender OR Age OR Education Background OR Occupation OR Location), the search was then narrowed by the category of law-abiding behavior and higher education sector using only the paper's title, keywords, and abstract. Based on a literature assessment of law-abiding behavior and/or the higher education sector, five more keywords were added to increase search relevancy and prevent missing relevant articles (Gender, Age, Education Background, Occupation, and Location).

### Research findings

The VOS viewer software is used to map the graphical representation of bibliographic data (Van Eck and Waltman, 2010). The VOSviewer converted the bibliographic data into graphs using the specified input data. A number of bibliometric methodologies were used to analyze the data, including BC, co-citation, and co-occurrence of the author's keyword. The citation BC is used when two authors “A” and “B” cite the third author's document “C.” Co-citation occurs when two publications are cited by a third document, such as when studies A and B are discussed in study C. Furthermore, keyword co-occurrence is computed by counting the number of times a phrase occurs in the same article (Van Eck and Waltman, 2017). In order to more precisely discover target publications, we performed a title search, which yielded a total of 3,785 articles due to the fact that the quantity of original material was too large and the results included numerous irrelevant literatures. Then, we manually eliminated the literature that did not focus the relationship between law-abiding behavior and higher education. After screening 2,500 articles were selected, covering a period from 2000 to end of July 2022 (see Figure 1).

The number of articles published between 2000 and the end of July 2022 is shown in Figure 1. According to the Figure 1, study on law-abiding behavior goes back to the year 2000, although it was not generally acknowledged until 2011. Since then, there has been a continuous increase in study into what law-abiding behavior is and how it enhance human civilization. Although, the law and morality are the foundations of human civilization (Steiner et al., 2008), and they are inextricably linked to the specific historical circumstances of a society's evolution and its existing social and class structure. Responsible law-abiding behavior is determined by a person's legal culture (Tyler and Darley, 1999) which is defined by a deliberate submission to the requirements of the law, compliance with social and moral standards, and adherence to rules of behavior. In a society where a legal culture has developed, people follow legal norms out of their own free will and in response to their own unique legal awareness (Khamidullaevna, 2022). Doing what the law requires is adhering to its intended purposes, guiding principles, and stipulations. This is, by definition, legal norm behavior, which takes many forms throughout society (activity, individual actions, legitimate inaction, verbal activity, which has legal significance). If a legal identity is developed that contains a set of values, norms, and rules that are executed voluntarily and on the basis of which an actually existent rule of law is constructed, then the normative social and legal action of the person will predominate. The development of children and youngsters' legal competence and legal awareness necessitates the cultivation of a favorable disposition toward the law on their part. The sudden drop in the number of articles published in 2020 may have been caused all higher education's institutes closed due to COVID-19 Pandemic. Moreover, in 2021 virtual classes were started, however, has not slowed down since 2021, and is therefore still a hot issue today.

Table 1 shows the top 20 journals, the maximum number of articles were published in Behavioral And Brain Sciences with 87 documents, 1850 citation and 88 Total link strength (TLS). Encyclopedia of Criminology and Criminal Justice is 2nd journal with 52 documents, 105 citation and 59 TLS. The three top citied journals are Behavioral and Brain Sciences, Journal of Business Ethics and Frontiers in Psychology. Top 20 journals are presented in the Table 1.

**Table 1:** No. of Publications based on Journals.

**No**.	**Journal name**	**Total link strength**	**Documents**	**Citations**
1.	Behavioral and brain sciences	88	87	1850
2.	Encyclopedia of criminology and criminal justice	59	52	105
3.	Encyclopedia of immigrant health	2	44	31
4.	Journal of business ethics	445	25	1382
5.	International journal of environmental research and public health	217	20	126
6.	Frontiers in psychology	248	19	417
7.	Plos one	142	16	246
8.	Accident analysis and prevention	184	15	305
9.	Frontiers of law in china	1	15	7
10.	Social learning theory and the explanation of crime	8	14	91
11.	International journal of offender therapy and comparative criminology	419	13	149
12.	Sustainability	178	12	148
13.	Asian journal of criminology	348	11	74
14.	International journal of urban and regional research	24	9	58
15.	Advances in intelligent systems and computing	2	7	3
16.	BMC public health	48	7	24
17.	China journal of social work	22	7	13
18.	Environmental science and pollution research	58	7	38
19.	Frontiers of education in china	13	7	15
20.	Journal of environmental management	16	7	194

**Figure 2:**
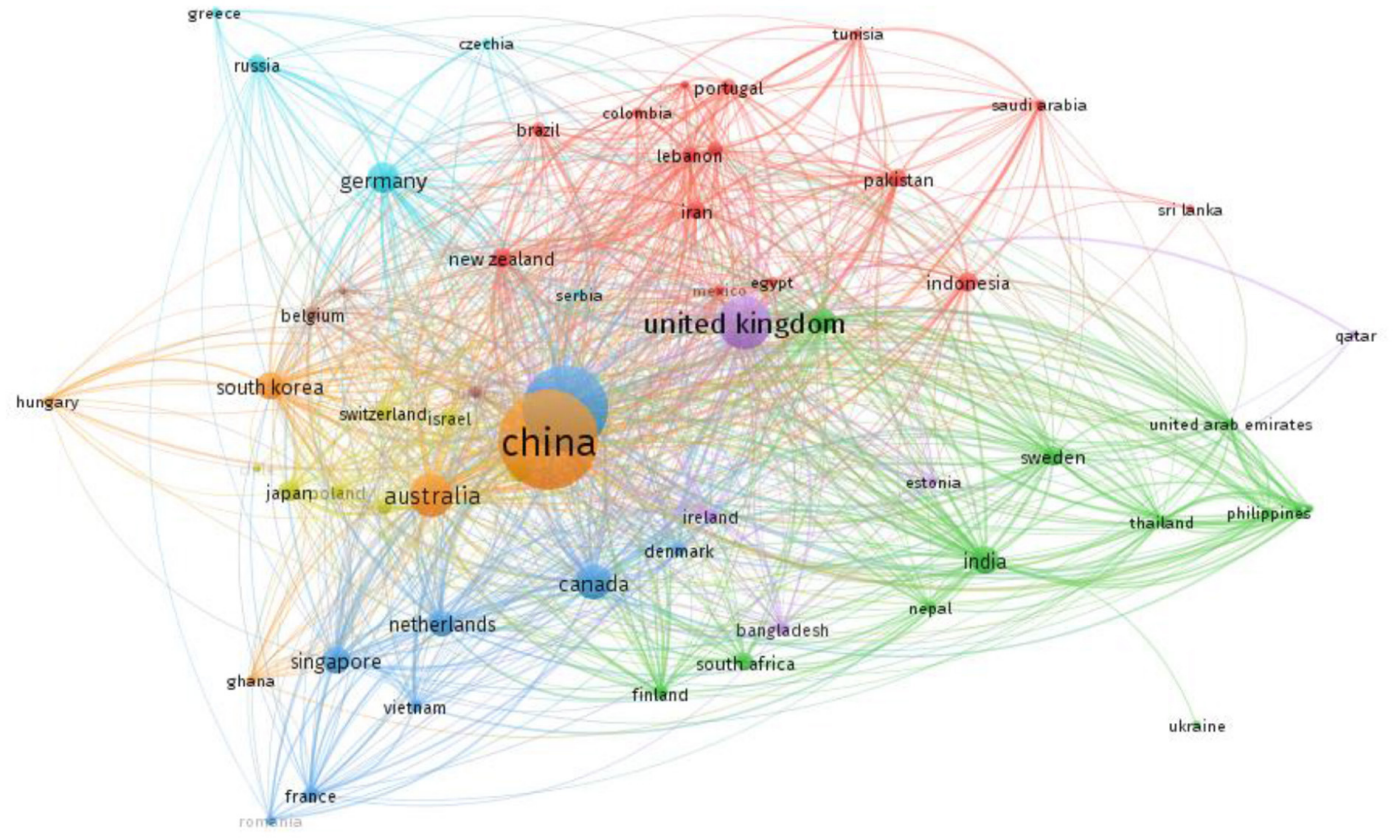
Articles published in term of country.

**Figure 3:**
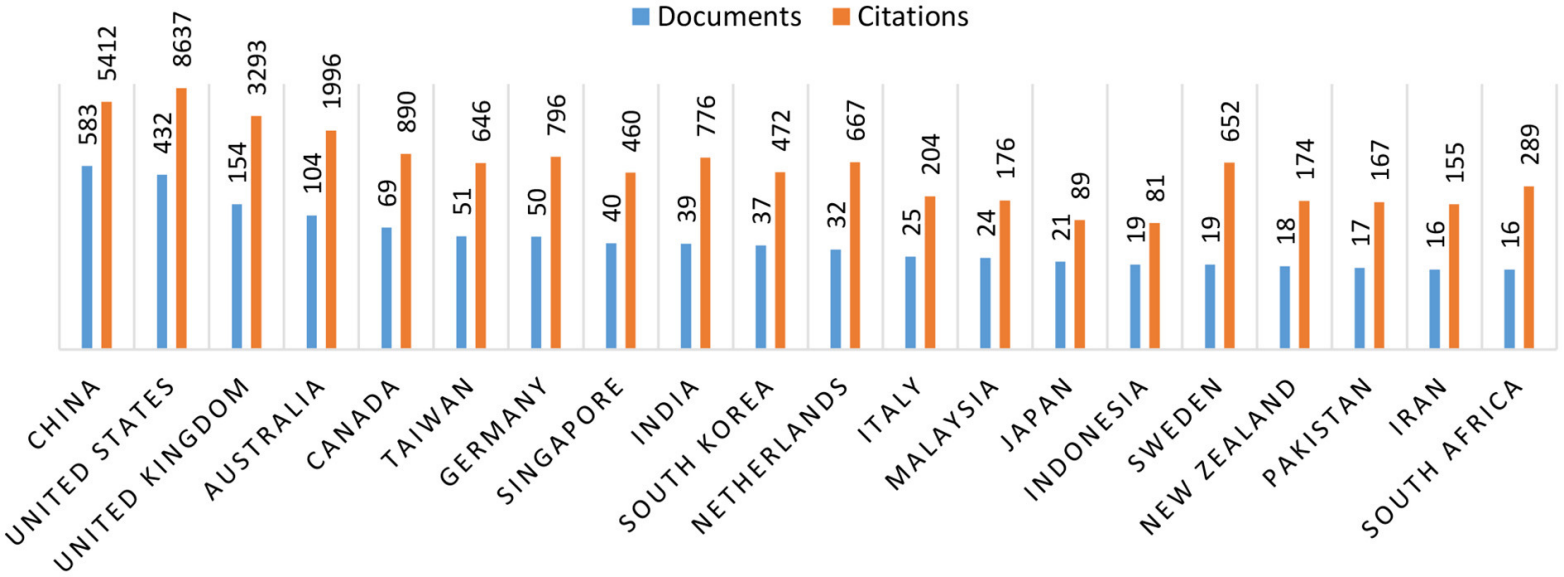
Number of documents and citations published in term of country.

Figure 2 shows the time span of published literatures in different countries. The literature was written by authors of multiple countries. If the maximum number of authors' nationality had two or more, we accepted all results for we thought the literature was created by different countries. It's shown that China led the analysis of law-abiding behavior in terms of number of articles (583 published and citations 5,412). Although, United Sates was leading in terms of citations (432 published and citations 8,637). However, only China has become the leader from Asian countries in law-abiding behavior study since 2000, which means research with background in China has become the hotspot. The remaining all in top 5 were developed countries United Stated (432 published and citations 8,637), United Kingdom (154 published and citations 3,293), Australia (104 published and citations 1,996), Canada (69 published and citations 890) (see Figure 3).

There are 5,123 authors that have published 2,500 articles on law-abiding behavior. On the basis of number of articles published, citations received, number of publications, H-index, and institutional affiliation, the 15 most prolific authors are shown in Table 2. The maximum number of articles published by Jiang, Shanhe and Keung, Hing also these authors get maximum citations 100 and 79, respectively.

**Table 2:** No. of publications and citations based on authors.

**No**.	**Author name**	**Total link strength**	**Documents**	**Citations**
1.	Jiang, Shanhe	707	8	100
2.	Keung, Hing	101	6	79
3.	Lambert, Eric G.	613	6	81
4.	Liu, Jianhong	827	6	45
5.	Boateng, Francis D.	776	5	22
6.	Cheng, Kevin Kwok-Yin	406	5	45
7.	Cheung, Chau-Kiu	297	5	28
8.	Chui, Wing Hong	350	5	32
9.	Li, Hui	65	5	39
10.	Shek, Daniel T. L.	701	5	13
11.	Wang, Wei	111	5	76
12.	Wu, Yuning	801	5	35
13.	Heberer, Thomas	4	4	39
14.	Ma, Hing Keung	241	4	71
15.	Rowley, Chris	52	4	29

The bibliographic coupling by organization the City University of Hong Kong is 1st organization with 25 documents, 292 citation and 1,913 Total link strength (TLS). Chinese University of Hong Kong is 2nd organization with 23 documents, 230 citation and 1297 TLS. The three top citied Organizations are University of Oxford, University of California, Los Angeles and Harvard University. Top 20 organization are list is blow in the Table 3.

**Table 3:** Bibliographic coupling by organization.

**NO**.	**Organization**	**Total link strength**	**Documents**	**Citations**
1.	City University of Hong Kong	1,913	25	292
2.	Chinese University of Hong Kong	1,297	23	230
3.	National University of Singapore	1,006	20	189
4.	Harvard University	2,461	18	1,439
5.	Tsinghua University	722	18	103
6.	Renmin University of China	806	17	112
7.	Hong Kong Baptist University	1,008	16	200
8.	Hong Kong Polytechnic University	1,065	16	136
9.	Nanyang Technological University	516	16	133
10.	Zhejiang University	1,267	16	307
11.	Australian National University	379	14	246
12.	Beijing Normal University	365	14	54
13.	Wayne State University	1,684	14	121
14.	University of California, Los Angeles	1,953	13	1,486
15.	University of Washington	990	13	524
16.	Sun Yat-sen University	923	12	63
17.	The University of Sydney	696	12	208
18.	University of Oxford	346	12	1,497
19.	University of Toronto	1,629	12	78
20.	East China Normal University	653	11	22

## Discussion

The current research presents bibliometric indicators of lawful behavior in the context of scientific research from the year 2000 to the end of July 2022. Using the Scopus database, this study analyzed more than 3,785 articles published on the topic of lawful behavior between 2000 and the end of July 2022. The original sample was then reduced to include only articles, book chapters, and conference papers that made significant contributions to the field of lawful behavior in the context of higher education. Our findings of reviewed articles concluded that more articles were published in the year of 2019 as compared to before and after year 2019. The current study emphasized that law and morality are the most important parts of human culture. They are always connected and depend on the specific historical circumstances of a society's development its social and class structure. Thus it is essential that students be knowledgeable about legal issues, orientated in terms of law-abiding behavior, aware of the nature of offences, and willing to bear the responsibility that is assigned to them (Maltseva et al., 2020). Furthermore, a law-abiding society, in which most people follow the law and obey legal authorities because they agree with the law and want to work with legal authorities, is better than one in which legal authorities have to threaten or use force to get people to obey. The foundation of every rule-abiding society is a political system in which its constituents have social values that compel individuals to take personal responsibility for avoiding rule violations, regardless of the risk of being discovered and penalized for their transgressions (Tyler and Darley, 1999).

Awareness of the law and following the rules are two factors that contribute to law-abiding conduct. The emergence of legal consciousness stems from people' abstract concepts of the equilibrium between desired order and peace. There is a strong association between legal awareness and other values, including those of a social, political, economic, and legal nature. In essence, law-abiding behavior can be noble principles that can affect all current systems, from the government's implementation of laws to the government's adoption of regulations to the government's administration of those rules (Asmah and Salam, 2022). For instance, previous studies have linked people not wearing safety equipment like seatbelts and helmets to an increased risk of car crashes (Zhou et al., 2022). In addition, as the popularization of Chinese law continues to develop, more and more Chinese citizens are becoming familiar with their legal rights and are able to evaluate whether or not those rights have been violated. However, some people, due to their preconceived notions about lawyers and the legal system, are unwilling to learn about the law, perceive legal procedures as difficult to operate, and avoid seeking legal assistance when their rights and interests are violated (Guan et al., 2022). Our study supports the conclusions of previously reviewed literature. The study investigates the significance of higher education in fostering law-abiding behavior among Chinese students and also highlights the impact of different factors on law obeying behavior. These elements include age, gender, education, occupation, and location. It has been shown in the literature that women are more attuned to lawful conduct than men. Likewise, previous study revealed that females may be more responsive to punishments for academic dishonesty than males, who are typically viewed as more competitive and risk-taking (Zhang et al., 2018). In a similar vein, there are other factors that have a significant link with lawful conduct. However, there has only been a little amount of research done on law abiding behavior up until now. So, it's suggested that scholars in the future look into lawful behavior in various population and across different countries.

### Future recommendations and implications

Following are some of suggestions. First, future researchers should gather longitudinal data to learn how students' knowledge and other factors affect their compliance with laws over time. Secondly, it would be helpful to do focus groups and interviews with the students, as the qualitative remarks are brief. Third, additional researches need to be conducted on the topic of law abiding behavior as previously limited studies were present. Fourth, future research should consider the influence of other factors on law-abiding behavior. Fifth, future research should conduct cross- cultural studies to compare the significance of law abiding behavior across different countries.

The study findings revealed that the rise of academic dishonesty like plagiarism in higher education highlights the importance of law abiding behavior among students (Shek et al., 2022). This area of research may aid universities in combating academic dishonesty through effective teaching and learning strategies that encourage a growth attitude rather than a fixed mindset regarding learning. So, for future studies, it is important to learn about law-abiding behavior and how it can help students who don't follow the law in the long run. The results also showed that those with greater education are more likely to respect authoritative figures and accept responsibility for their conduct (Wu et al., 2022). In this way, future researchers should stress how important education is for students because it gives them not only knowledge and skills, but also spiritual and moral values that shape who they are and bring people together. In the future, researchers should use effective methods to figure out the complicated internal and external factors that lead to cheating and other bad behavior in higher education. This would lead to better results. As a result, effective institutional and programmatic interventions may concentrate on modifying students' mindsets in order to combat the disruptive conduct and improve educational outcomes.

Our study findings would be beneficial for the academic researchers and policy makers in following ways. First in educational institutions precautionary measures, such as providing incoming students with detailed information on policy requirements and learning resources about academic integrity in advance of formal registration, may help them to comprehend and adjust to the academic dishonesty. One way to put this into practice is for teachers to adjust their methods of instruction to make cheating less appealing. For example, if you make sure the course content is based on what the students ask and the assignments are frequent, short, and realistic, the students will gain confidence and won't need to cheat. Second, students in higher education institutions need to learn about obedience and the law in order to maintain a caring attitude towards the nation and state, which is essential for the development of a humane character. Third, one strategy of preventing criminal behavior is teaching lawful values to college and university students. Fourth, participating in religious events can reawaken students to maintain behavior consistent with their religious norms. Fifth, honesty also discourages students from acting dishonestly in class, which can prevent them from being manipulative. Lastly, every student needs to adopt the mindset that discipline is valuable in order to encourage compliance, maturity, and responsibility in the context of academic norms.

## Conclusion

In this study a total of 3,785 articles were searched from 2000 to the end of July 2022 years on VOS viewer software. This study focuses to answer following four questions: What is law abiding behavior? What factors affect the higher education student's awareness of law-abiding in China? Which factors have more significant impact on law abiding of higher education students? Is there any relationship between law abiding behavior and psychological factors in higher education students? Based on the reviewed articles, it was found that in the year 2019, more articles were published on law abiding behavior as compared to before and after year 2019.

Present study focuses on the impact of different factors such as age, gender, education, occupation, and location on law-abiding behavior. The conclusion of various literature supports that there is a significant impact of these factors on law abiding behavior. Our findings not only shed light on the aspects that are most important in shaping law-abiding behavior, but they also imply that the educational system as a whole should implement initiatives to encourage law-abiding behavior among its students. To achieve this objective, it is vital to establish a climate of respect in the university that precludes any disorganization or deviant behavior on the part of students, such as violence, drug use, and addictive behavior etc. Moreover, assessment of social credit will lead to more law-abiding people in the community and, as a result, improve the quality of society as a whole. For a country to prosper economically and socially, it must have trustworthy and law-abiding individuals.

## Data availability statement

The raw data supporting the conclusions of this article will be made available by the authors, without undue reservation.

## Author contributions

All authors listed have made a substantial, direct, and intellectual contribution to the work and approved it for publication.

## Funding

This work was supported by Major Project of China Social Science Foundation: Carry Forward the Spirit of Socialist Rule of Law (22ZDA072).

## Conflict of interest

The authors declare that the research was conducted in the absence of any commercial or financial relationships that could be construed as a potential conflict of interest.

## Publisher's note

All claims expressed in this article are solely those of the authors and do not necessarily represent those of their affiliated organizations, or those of the publisher, the editors and the reviewers. Any product that may be evaluated in this article, or claim that may be made by its manufacturer, is not guaranteed or endorsed by the publisher.
